# Diagnostic and prognostic value of presepsin in the management of sepsis in the emergency department: a multicenter prospective study

**DOI:** 10.1186/cc12847

**Published:** 2013-07-30

**Authors:** Marco Ulla, Elisa Pizzolato, Manuela Lucchiari, Maria Loiacono, Flavia Soardo, Daniela Forno, Fulvio Morello, Enrico Lupia, Corrado Moiraghi, Giulio Mengozzi, Stefania Battista

**Affiliations:** 1 Emergency Medicine Department, "Città della Salute e della Scienza" University Hospital, Corso Bramante 88, I-10126 Turin, Italy; 2 Clinical Biochemistry Laboratory, "Città della Salute e della Scienza" University Hospital, Corso Bramante 88, I-10126 Turin, Italy; 3 Emergency Medicine Department, Imperial College NHS Trust, St Mary's Hospital, Praed Street, London, W2 1NY, UK

## Abstract

**Introduction:**

Sepsis, severe sepsis and septic shock are common conditions with high mortality. Their early diagnosis in the Emergency Department (ED) is one of the keys to improving survival. Procalcitonin (PCT) has been used as a biomarker in septic patients but has limited specificity and can be elevated in other scenarios of systemic inflammatory response syndrome (SIRS). Soluble CD14 (sCD14) or presepsin is the free fragment of a glycoprotein expressed on monocytes and macrophages. Preliminary reports suggest that levels of presepsin are significantly higher in septic patients than in healthy individuals. The aim of this study is to investigate the diagnostic and prognostic value of presepsin compared to PCT in people presenting at the ED with SIRS and suspected sepsis or septic shock.

**Methods:**

This study was conducted in two major hospitals in Turin, Italy. One hundred six patients presenting to the EDs with suspected sepsis or septic shock were included, and another eighty-three patients affected by SIRS, but with no clinical evidence of infection, were recruited as controls. Blood samples were collected at first medical evaluation and for some patients after 24 and 72 h. The samples were analyzed using the PATHFAST Presepsin assay for sCD14, and commercial kits were used for other determinations (for example, PCT). Definitive diagnosis and survival rates were obtained afterward by analysis of digital medical records.

**Results:**

Elevated concentrations of presepsin at presentation were observed in septic patients compared to control patients. The same trend was observed for mean values of PCT. Higher values of presepsin were observed in septic patients at presentation (time 0). The diagnostic accuracy of PCT was generally higher, and areas under the curve (AUCs) were 0.875 for PCT and 0.701 for presepsin. Mean presepsin values were significantly higher in nonsurvivor septic patients (60-day mortality) than in survivors. No significant correlation was noted between PCT and survival.

**Conclusions:**

In our experience, presepsin was useful in the early diagnosis of infection in a complex population of patients with SIRS, sepsis, severe sepsis and septic shock who presented to the ED. Presepsin showed a significant prognostic value, and initial values were significantly correlated with in-hospital mortality of patients affected by sepsis, severe sepsis or septic shock.

## Introduction

Sepsis, severe sepsis and septic shock are some of the most common conditions handled in the Emergency Department (ED) and ICU, and, despite modern antibiotic therapy in conjunction with cardiovascular and respiratory support, mortality rates remain between 30% and 60% [1-3]. According to the most recent guidelines, published in 2013 by the Surviving Sepsis Campaign, early recognition of these conditions and the speed and appropriateness of therapy in the initial hours after presentation are likely to influence the outcomes of septic patients [4].

More recently, the biomarkers used as diagnostic criteria for sepsis, plasma C-reactive protein (CRP) or procalcitonin (PCT) levels more than 2 standard deviations (SD) above the normal value, are now part of the inflammatory variables which, together with infection, whether documented or suspected, constitute a definition of sepsis [4,5]. There is also good evidence that low PCT levels or similar biomarkers can be used to assist the clinician in the critical care areas in the discontinuation of empiric antibiotics in those patients who appear septic, but have no subsequent evidence of infection [6]. Although PCT has an established role as a biomarker in septic patients and has been shown to correlate closely with infection, it has some limitations. It rises transiently in patients with nonseptic conditions and systemic inflammatory response syndromes (SIRS) (for example, trauma, surgery, heatstroke) and is not detectable in certain cases of sepsis. Furthermore, biological predictors of mortality are absent, clinical scores appear to be of limited value and the role of PCT as a poor prognostic factor in patients admitted to the ED because of sepsis remains to be proved [7]. The ideal biomarker should retain high sensitivity and specificity and be cost-effective and promptly available.

Cluster of differentiation 14 (CD14) is a glycoprotein expressed on the membrane surface of monocytes and macrophages and serves as a receptor for complexes of lipopolysaccharides (LPSs) and LPS-binding proteins (LPBs). By activating a proinflammatory signaling cascade on contact with infectious agents, CD14 has a role as a pattern recognition molecule in the innate immune response against microorganisms [8]. During inflammation plasma protease activity generates soluble CD14 (sCD14) fragments. One of them, called sCD14 subtype (sCD14-ST), or presepsin, has recently been identified. Presepsin is normally present in very low concentrations in the serum of healthy individuals and has been shown to be increased in response to bacterial infections according to the severity of disease. Moreover, rapid dosage methods, based on chemiluminescence enzyme immunoassay, are available and allow automated measurements in a short time [9,10]. Preliminary studies suggest that the level of presepsin significantly differs in healthy individuals and in patients with local infection, SIRS, sepsis or severe sepsis [11,12]. Presepsin is currently under investigation in clinical practice as a reliable marker of adult and neonatal sepsis [13,14] and for the postmortem diagnosis of sepsis-related death [15].

On the basis of these premises, we designed a multicenter prospective study to investigate the diagnostic role and efficacy, together with prognostic power, of presepsin, compared to PCT in an adult population presenting to the ED with SIRS (control group) or suspected sepsis or septic shock (study group).

## Patients and methods

### Patients and design of the study

We conducted a multicenter prospective study of patients presenting to the ED who met at least two criteria for SIRS and in whom sepsis was suspected to be the primary or concurrent diagnosis. Informed consent to participate in the study was obtained from the patients or their families, and the study was approved by the Ethics Committee of Turin University Hospital according to the Declaration of Helsinki (1964).

The duration of the study was 1 yr. Between January 2012 and January 2013, 189 patients presenting to the EDs of San Giovanni Battista University Hospital and Major Trauma Center Hospital, both in Turin, Italy, were enrolled. The inclusion criteria were the presence of at least two clinical criteria for SIRS; suspected diagnosis of sepsis, severe sepsis or septic shock (according to the guidelines of the American College of Chest Physicians/Society of Critical Care Medicine and the International Surviving Sepsis Campaign Guidelines Committee) [16,17]; and patients who could undergo a blood test. Exclusion criteria were age less than 18 years old, incapability to give informed legal consent and evidence of a clearly different diagnosis.

One hundred six patients (43 females, 63 males) presented at the ED with suspected sepsis or septic shock and 83 more patients (30 females, 53 males) affected by SIRS were recruited as controls. In Table 1, the demographic and clinical characteristics of the patients enrolled in the study are summarized. Clinical and biochemical data were recorded on admission. Blood samples were collected at the time of first medical evaluation (time 0, T0) in the ED before any medical treatment. Initial Sequential Organ Failure Assessment (SOFA) score [18] and Acute Physiology and Chronic Evaluation II (APACHE II) score [19] were calculated in the ED using clinical parameters and blood test results. After the first evaluation in the ED, patients were admitted in intensive care areas (high-dependency unit, ICU or monitored medical ward). A few patients were later lost at follow-up; among the rest of them, blood specimens were collected after 24 hours (time 1, T1) and 72 hours (time 2, T2) to study the temporal trend of biomarkers. All the samples were stored at -70°C and thereafter analyzed blindly for sCD14-ST (presepsin) and PCT in the Clinical Chemistry Laboratory of Turin University Hospital. The definitive diagnosis (SIRS, sepsis, severe sepsis or septic shock) was made according to the criteria of the International Guidelines for Management of Severe Sepsis and Septic Shock [4,16] and afterwards obtained by clinicians by analysis of digital medical records (Table 2). We evaluated survival on the basis of 60-day in-hospital mortality, and survival rates were then blindly matched to biomarker values obtained at the first evaluation in the ED.

**Table 1:** Demographic and clinical characteristics of the subjects in the study.^a^

	SIRS (*n *= 83)	Sepsis (*n *= 55)	Severe sepsis/septic shock (*n *= 51)
Male	53	32	31

Female	30	23	20

Age	56 (19 to 92)	71 (27 to 99)	71 (22 to 90)

SOFA score	2 (0 to 8)	3 (0 to 9)	5 (0 to 15)

APACHE II score	7 (0 to 19)	11 (2 to 26)	14 (4 to 33)

^a^APACHE II: Acute Physiology and Chronic Evaluation II; SIRS: systemic inflammatory response syndrome; SOFA: Sequential Organ Failure Assessment. Values are means and minimum and maximum range. SOFA and APACHE II scores reported were calculated at the very first evaluation in the Emergency Department.

**Table 2:** Definitive diagnosis and infective foci of patients included in the study.^a^

SIRS (n = 83)			Sepsis (*n *= 55)	Severe sepsis/septic shock (*n *= 51)
Acute pancreatitis	18 (22%)	Urinary tract	14 (25%)	13 (25%)
Aortic dissection	15 (19%)	Lung	43 (78%)	30 (59%)
Pulmonary embolism	2 (2%)	Abdomen	11 (20%)	9 (18%)
Acute coronary syndrome	16 (19%)	Others	8 (14%)	4 (8%)
Major trauma	27 (32%)			
Burns	5 (6%)			

^a^SIRS: systemic inflammatory response syndrome. Others: central venous catheter, central nervous system and skin. Values are expressed as absolute numbers and percentages. Twenty-one patients in the sepsis group and five patients in the severe sepsis/septic shock group had more than one infection source.

### Measurement methods

Blood samples were collected in endotoxin-free tubes containing ethylenediaminetetraacetate (EDTA), centrifuged at 3,000 *g *for 10 min and then stored at -70°C until being assayed blindly for the presepsin and PCT measurements. Presepsin was dosed using the PATHFAST Immunoanalyzer system (Mitsubishi Chemical Europe GmbH, Düsseldorf, Germany), based on noncompetitive chemiluminescence enzyme immunoassay for the quantitative measurement of the biomarker concentration in anticoagulated (heparin or EDTA) whole blood or plasma. The PATHFAST Presepsin kit (Mitsubishi Chemical Europe GmbH) includes cartridges composed of 16 wells, containing all needed reagents, that is, magnetic particles coated with mouse monoclonal antibodies against presepsin, alkaline phosphatase (ALP)-labeled rabbit polyclonal antibodies, chemiluminescent substrate, incubation buffers and washing solutions. Presepsin in the specimen binds to the anti-presepsin antibodies to form an immunocomplex with the ALP-labeled antibodies and the antibody-coated magnetic particles. After removal of the unbound ALP-labeled antibodies using MAGTRATION Technology (magnetic particles are directly washed in the dispensing cap; Precision System Science Europe GmbH, Wörrstadt, Germany), a chemiluminescent substrate is added to the immunocomplex. After a 10-min incubation period, the luminescence generated by the enzyme reaction is detected by a photomultiplier to calculate the concentration of presepsin in the samples using the measurement of light emission. The assay takes 17 min using a sample volume of 100 µl. The entire procedure was automatically performed using the PATHFAST Immunoanalyzer (Mitsubishi Chemical Europe GmbH). Method precision was tested on three different plasma pools with a mean presepsin concentration of 327 pg/ml (range, 700 to 7,201 pg/ml), yielding within-series coefficients of variation (CVs) of 12.3%, 3.9% and 2.8%, respectively, as well as between-series CVs of 15.0%, 7.7% and 4.2%, respectively.

Quantitative analysis of PCT was performed using an automated electrochemiluminescence immunoanalyzer (ELECSYS* BRAHMS* PCT; Roche Diagnostics, Mannheim, Germany). The assay time was 18 min and was automatically performed on Cobas e601 analyzer platforms (Roche Diagnostics). According to our experience, within-run and between-run precision for this test were 5.6% and 8.8%, respectively, at 0.15 ng/ml PCT concentration, 4.2% and 5.7% at 1.7 ng/ml, and 2.5% and 4.9% at 20.3 ng/ml, respectively.

### Statistical analysis

The Kruskal-Wallis test with the Bonferroni correction was used to explore the results in each group (SIRS, sepsis and severe sepsis or septic shock) at the first presentation to the ED, to examine the capability of the two biomarkers to diagnose sepsis, to analyze the different primary sites of infection and to compare the different populations in the control group (acute pancreatitis, aortic dissection, pulmonary embolism, acute coronary syndrome, multiple trauma and burns). The Mann-Whitney *U *test was applied to evaluate efficacy regarding 60-day mortality prediction and to analyze the diagnostic significance in the different populations in the group of patients with no infection. Correlations between biomarkers and blood test results, biomarkers and SOFA and APACHE II scores were analyzed using Spearman's rank correlation test. The Friedman test with the Bonferroni correction was used to study the laboratory trend of biomarkers from T0 to T2. A *P *value less than 0.05 was considered to be significant. Statistical tests were performed using MedCalc version 10.0.1.0 statistical software (MedCalc Software, Ostend, Belgium) and Analyse-it Standard Edition 3.02 software (Analyse-it Software, Ltd, Leeds, UK) for Microsoft Excel (Microsoft, Redmond, WA, USA).

## Results

### Patient characteristics

A total of 189 patients were included in the study. Eighty-three of them presented with SIRS due to acute medical conditions or trauma and were enrolled in the control group. Fifty-five patients were diagnosed with sepsis, and fifty-one had severe sepsis or septic shock. Demographic characteristics and mean SOFA and APACHE II scores at presentation are reported in Table 1.

### Diagnostic role

**Figure 1:**
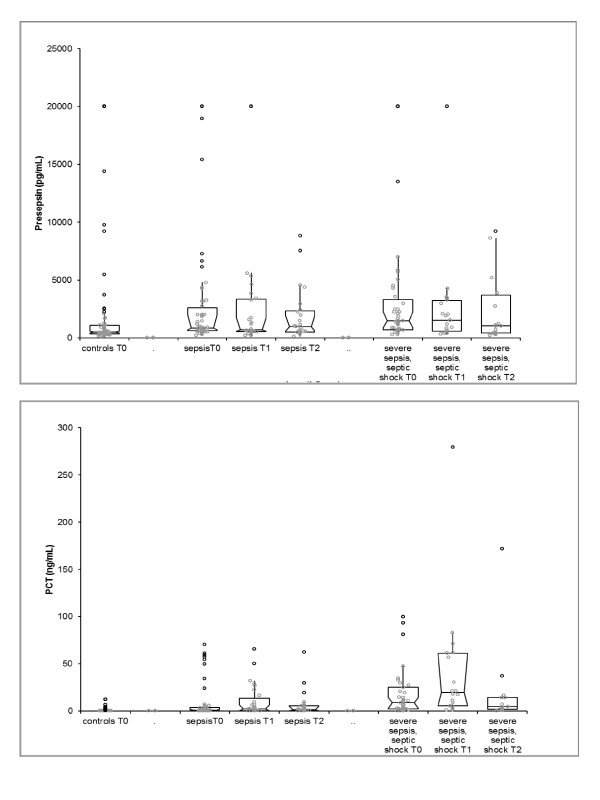
**Median (boxplots) values of presepsin and procalcitonin in controls and in sepsis and severe sepsis/septic shock patient populations at time 0. 95% confidence intervals (CIs) are reported**. Median (boxplots) seriated values of presepsin and procalcitonin (PCT) in 42 patients with sepsis and severe sepsis/septic shock at time 0 (T0), T1 and T2. 95% CIs are reported. Presepsin displayed highest concentration at T0 (*P *= 0.0444) compared to T1 and T2. The highest PCT levels were observed at T1 (*P *< 0.0001) compared to T0 and T2.{AU: Please define gray and black circles in figure legend. Gray circles represent values of presepsin or procalcitonin included between the min and the greatest value excluding outliers; black circles represent outliers. }

The mean presepsin concentrations in plasma were 2,516.4 pg/ml in the control group, 2,866.7 pg/ml (range, 1,579 to 4,154) in patients affected by sepsis and 3,167.1 pg/ml in the severe sepsis and septic shock group. Mean levels of PCT in controls, sepsis and severe sepsis and septic shock patients were 1 ng/ml, 9 ng/ml and 19 ng/ml, respectively. Median values for presepsin were 517 pg/ml in controls, 875 pg/ml in septic patients and 1,460 pg/ml in patients with severe sepsis or septic shock (Figure 1). Presepsin initial values (T0) were significantly different between controls and the sepsis group (*P *= 0.002) and between controls and the severe sepsis and septic shock group (*P *< 0.001), whereas no significant difference was noted between the sepsis and severe sepsis/septic shock groups (*P *= 0.07). PCT values at presentation (T0) were conversely significantly different between all three groups (Table 3).

**Table 3:** Mean values and (95% confidence intervals) of presepsin and procalcitonin in the three populations.^a^

	SIRS	Sepsis		Severe sepsis/septic shock	
	
	Mean (95% CI)	Mean (95% CI)	*P *value	Mean (95% CI)	*P *value
Presepsin pg/ml (T0)	2,516.4 (1,360.3 to 3,672.4)	2,866.7 (1,579 to 4,154)	vs controls: 0.02 vs severe s/ss: 0.07	3,167.1 (1,686.5 to 4,647.7)	vs controls: <0.001 vs sepsis: 0.07

PCT ng/ml (T0)	1 (0 to 1)	9 (4 to 14)	vs controls: <0.001 vs severe s/ss: 0.005	19 (10 to 28)	vs controls: <0.001 vs sepsis: 0.005

^a^CI: confidence interval; PCT: procalcitonin; SIRS: systemic inflammatory response syndrome. *P *values depict correlation between different populations. s/ss: severe sepsis/septic shock.

Both presepsin and PCT concentrations were significantly lower in all nonseptic SIRS patients (acute pancreatitis, aortic dissection, pulmonary embolism, acute coronary syndrome, multiple trauma and burns) in comparison to patients with sepsis, severe sepsis or septic shock. Presepsin levels at T0 significantly correlated with PCT levels at T0 (*P *= 0.001) and with SOFA score (*P *= 0.008). PCT levels at the time of the first medical evaluation in the ED were significantly correlated with high values of serum lactate (*P *= 0.004).

Presepsin and PCT initial values (T0) did not differ significantly according to the primary sites of infection (urinary tract, lung, abdomen, skin, central venous device-related, central nervous system (CNS) and oral infection) among patients with a confirmed diagnosis of sepsis, severe sepsis or septic shock when the starting focus could be identified (Table 4).

**Table 4:** Biomarkers blood levels according to primary sites of infections in patients with diagnosis of infection (sepsis, severe sepsis or septic shock) at time 0.^a^

	Urinary tract	Lung	Abdomen	Other sites	*P *value
	**Mean (95% CI)**				

Presepsin (pg/ml)	2,791.4 (1,032.6 to 4,550.2)	3,818.8 (2,434.5 to 5,291)	3,667.1 (607.1 to 6,746.7)	6,542.2 (1,141.1 to 11,047.4)	0.94

PCT (ng/ml)	17.13 (6.3 to 27.9)	6.37 (5.6 to 17.5)	14.5 (1 to 28.1)	10.6 (0.2 to 21.1)	0.29

^a^CI: confidence interval; PCT: procalcitonin. Other sites correspond to central nervous system, central venous catheter-related, skin and oral infections.

Serial blood tests were obtained for 42 patients to evaluate the temporal trend of presepsin and PCT. Among patients with a definitive diagnosis of infection, measurements obtained from monitoring biomarker values at T0 (first medical evaluation in the ED), T1 (24 hours after admission) and T2 (72 hours after admission) showed significant results. Presepsin displayed the highest concentration at T0 compared to T1 and T2, with a significant increasing trend over time (*P *= 0.0444). The highest PCT concentrations were observed after 24 hours (T1) in comparison to T0 and T2 (*P *< 0.001) (Figure 1).

### Diagnostic accuracy

**Figure 2:**
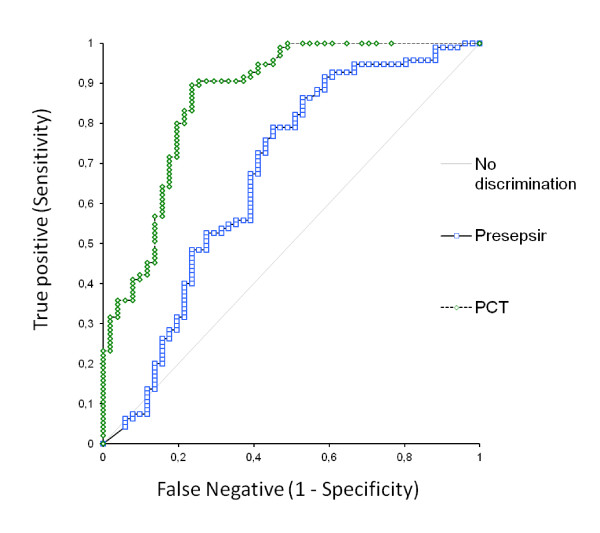
**Receiver operating characteristic curve for presepsin (blue) and procalcitonin (green) in patients with definitive diagnosis of sepsis, severe sepsis or septic shock**. Areas under the curve were 0.70 for presepsin (*P *< 0.001) and 0.87 for procalcitonin (*P *< 0.001).{AU: Please fix misspelling "Presepsina" to "Presepsin" in figure. See attached file for figure 1}

The receiver operating characteristic (ROC) curves were designed including those patients with a definitive diagnosis of sepsis or severe sepsis/septic shock (Figure 2). The results were significant for both biomarkers. The areas under the curve (AUCs) calculated from the ROC curve were 0.701 (95% confidence interval (CI), 0.63 to 0.77; *P *< 0.001,) for presepsin and 0.875 (95% CI, 0.82 to 0.92; *P *< 0.001) for PCT, respectively. The difference between the two AUCs was significant (*P *< 0.001). The best diagnostic cutoff for presepsin was 600 pg/ml. At that level, sensitivity and specificity were 78.95% (95% CI, 69.4 to 86.6) and 61.90% (95% CI, 50.7 to 72.3), respectively. The best diagnostic cutoff in terms of sensitivity and specificity for PCT was 0.18 ng/ml, corresponding to 89.47% sensitivity (range, they actually were 95% CI 81.5% to 94.8%) and 75.90% specificity (range, 65.3% to 84.6%).

### Prognostic role

**Figure 3:**
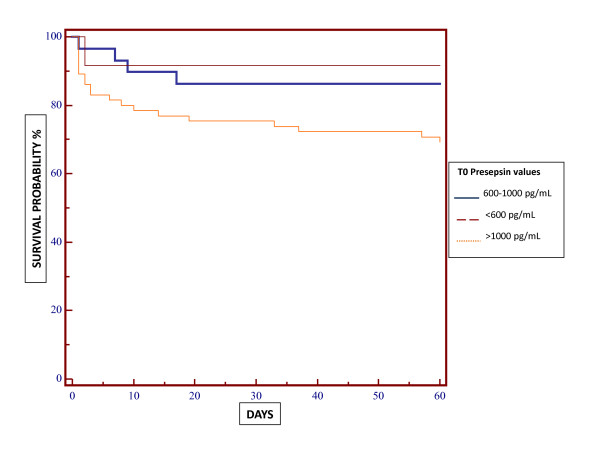
**Kaplan-Meier graph showing correlation between initial values (time 0) of presepsin and survival in patients with sepsis, severe sepsis and septic shock**. Sixty-day in-hospital mortality was higher in patients with initial values of presepsin greater than 1,000 pg/ml than in groups with lower values (*P *= 0.04).

Some patients were lost at the time of 60-day follow-up, but data for the remaining group were significant and representative (*N *= 118). A Kaplan-Meier graph showed that presepsin values at T0 (first evaluation in the ED) were correlated with 60-day in-hospital mortality in patients with sepsis and severe sepsis or septic shock (Figure 3). Mean initial levels were 4,232.4 pg/ml in nonsurvivor patients and 3,451.2 pg/ml in survivor patients (*P *= 0.04). No significant correlations were observed between presepsin level at T1 or T2 and survival rates. The comparison between PCT levels at T0 in nonsurvivor patients (17.12 ng/ml) and survivors (9.59 ng/ml) did not demonstrate a significant correlation (*P *= 0.87).

## Discussion

Sepsis is a systemic host response to infection and can in some conditions lead to severe sepsis (documented or suspected infection in addition to organ failure or dysfunction) or septic shock (severe sepsis and hypotension not responsive to fluid therapy). Severe sepsis and septic shock are frequently handled in the ED, and, despite modern antibiotic therapy, mortality rates remain high [20-23].

Early recognition of sepsis is not always straightforward, and the clinical signs themselves can be misleading, especially in patients with several comorbidities or variable demographic characteristics (age, sex, ethnic group), such as those who are normally admitted to the ED. Biomarkers, which were recently introduced among the inflammatory variables in the diagnostic criteria for sepsis [4], could contribute to prompt identification of those patients affected by sepsis, severe sepsis and septic shock who could benefit from quick and appropriate therapy.

Among different molecules which have been suggested as sepsis biomarkers in recent years, presepsin, or sCD14, appears quite promising on the basis of its reported correlation with the early stages of the septic process [9-15].

We designed a multicenter prospective study to validate the diagnostic and prognostic role of presepsin, compared to PCT, in the complex setting of the ED. Patients were recruited during the course of 1 yr in the EDs of two of the main university hospitals in Turin, Italy. Patients presenting with at least two clinical characteristics of SIRS were enrolled, definitive diagnoses were made according to the criteria of the International Guidelines for Management of Severe Sepsis and Septic Shock [4], and, according to the retrospective analysis of the clinical records, the population was divided into three groups: a control group (patients affected by SIRS due to acute pathologies such as acute pancreatitis, aortic dissection, pulmonary embolism, acute coronary syndrome, major trauma or burns, but with no evidence of infection), patients with sepsis (SIRS criteria together with documented evidence of infection) and severe sepsis or septic shock (sepsis and evidence of organ dysfunction or failure or unresponsive hypotension). Severe sepsis and septic shock were considered a unique group because of the similar clinical and prognostic features of these conditions [24]. The three groups in the study were homogeneous in terms of size and demographic characteristics.

Presepsin levels at presentation (T0) were significantly higher in patients affected by sepsis and severe sepsis/septic shock than in control patients. These data strongly support the value of this biomarker in the differential diagnosis of infection when used in the difficult case mix of patients with SIRS presenting to the ED. No difference in presepsin levels was found between the sepsis and severe sepsis groups, suggesting that the concentration of the biomarker is not related to the severity of disease in the very first hours. The PCT results in our study population confirmed the high diagnostic accuracy of this biomarker for sepsis recognition as well as its correlation with the severity of the disease.

Subpopulation analysis showed no significant correlation between presepsin results and the different populations in the control group (acute pancreatitis, aortic dissection, pulmonary embolism, acute coronary syndrome, major trauma and burns). A significant correlation was found between presepsin and PCT concentrations at T0 and between presepsin levels and SOFA score as a severity index of organ failure, strengthening our hypothesis that presepsin is specifically increased in patients with more severe infection.

Presepsin concentrations did not correlate significantly with the primary site of infection (urinary tract, lung, abdomen, skin, central venous catheter-related, CNS or oral), when it was possible to identify them using clinical, radiological or microbiological methods. This was probably due to the fact that presepsin, as with PCT, is produced systemically by circulating cells, not within a specific organ or body area.

The role of the new biomarker in monitoring septic disease was investigated by the analysis of serial measurements, which were performed at T0 (first medical evaluation in ED), T1 (24 h after admission) and T2 (72 h after the admission). Presepsin values were significantly higher at T0 than at T1 and T2, whereas PCT values were higher at T2. These results suggest that presepsin is an early biomarker for the diagnosis of sepsis and could play an important role in monitoring of the disease. This decreasing trend could be related to early clearance or to reduced production as a consequence of the appropriate treatment in the initial hours, affecting the systemic and unregulated immune response of the organism during sepsis. The role of PCT in monitoring the severity of disease is well-established and can assist the clinician in deciding about treatment [5,6].

The ROC curves were significant for both biomarkers, but the AUC calculated for PCT was wider, demonstrating a better diagnostic accuracy than presepsin. This evidence conflicts with what was previously reported by other groups [9-12], probably due to different criteria in selecting the study population. We enrolled a more complex and heterogeneous population of patients presenting to the ED with SIRS and several comorbidities, thus representing a true, "real-life" scenario. In our experience, the best diagnostic cutoff for presepsin, resulting from the ROC curve, was 600 pg/ml, which was similar to that previously reported by other groups. The complexity of our population case mix in terms of age, comorbidities and enrollment criteria is further supported by the optimal cutoff value calculated for PCT (0.18 ng/ml), which differs from that currently used in clinical practice (>2 ng/ml corresponding to high probability of sepsis).

Analysis of 60-day mortality showed the superiority of presepsin compared to PCT. Presepsin concentrations at the first evaluation in the ED were higher in nonsurvivor septic patients than in survivors. No significant correlation was found between PCT values and 60-day mortality. These results pointed out the possible prognostic role of presepsin in predicting in-hospital mortality and promptly identifying high-risk patients.

To the best of our knowledge, this study is the first to address the potential value of presepsin in the clinical scenario of the ED of two tertiary referral university centers. Our study population closely resembles the one ED physicians face in their daily practice, where the application of laboratory biomarkers may help them to pick up septic patients with a more severe prognosis to promptly start the appropriate treatment in the early hours after presentation. In this setting, presepsin has proven to be a promising new biomarker that is readily available, cost-effective and able to distinguish septic patients in a complex population presenting to the ED with SIRS. The close correlation between presepsin initial values and in-hospital mortality suggests its potential usefulness for the early recognition of high-risk septic patients, who could benefit from a more aggressive approach starting in the ED.

## Conclusions

Presepsin is a promising new biomarker that is readily available, cost-effective and able to distinguish septic patients in a complex population presenting to the ED with SIRS. Our experience suggests that presepsin can be used in the ED to promptly identify patients with SIRS due to severe infection.

The close correlation between presepsin initial values and in-hospital mortality suggests that this biomarker could be used to perform an early and reliable risk stratification and to identify high-risk patients who could benefit from a more aggressive approach starting in the ED.

## Key messages

• Presepsin is a new biomarker with good specificity and sensitivity in identifying patients with sepsis-related conditions in the ED.

• Combined use of presepsin and PCT can improve diagnostic accuracy for sepsis-related conditions in the ED.

• Presepsin has been shown to retain a prognostic role and closely correlate with in-hospital mortality of patients with severe sepsis and septic shock.

## Abbreviations

ALP: alkaline phosphatase; APACHE II: Acute Physiology and Chronic Evaluation II; AUC: area under the curve; CD14: cluster of differentiation 14; ED: Emergency Department; EDTA: ethylenediaminetetraacetate; LBP: lipopolysaccharide-binding protein; LPS: lipopolysaccharide; PCT: procalcitonin; ROC: receiver operating characteristic; sCD14-ST: soluble CD14 subtype; SIRS: systemic inflammatory response syndrome; SOFA: Sequential Organ Failure Assessment; T0-1-2: time 0-1-2

## Conflicts of interest

All the authors declare no conflicting interests, financial support or nonfinancial/academic interests. Presepsin commercial kits were provided by Mitsubishi Chemical Medience Corporation without any payment. PCT was tested as part of a routine assay performed in the emergency departments with no financial support.

## Authors' contributions

MU and EP enrolled the patients; acquired, analyzed and interpreted data; and wrote the manuscript. MLu and MLo managed plasma samples and performed biochemical assays. FS, DF, FM, EL and CM enrolled patients and contributed to data analysis and interpretation. GM and SB designed and coordinated the research, analyzed and interpreted data and reviewed the manuscript. All authors read and approved the manuscript for publication.
